# A System of Gynecology, by American Authors

**Published:** 1887-11

**Authors:** 


### Book Reviews.

---

**A System of Gynecology, by American Authors.** Edited by Mathew D. Mann, M. D., Professor of Obstetrics and Gynecology in the Medical Department of the University of Buffalo. Vol. I. Illustrated with three colored plates and two hundred and one engravings on wood. Philadelphia: Lea Bros. & Co. 1887.

This work is worthy of the commendation of the profession, which it has already received from so many sources. The contributors are all men of distinction in the profession. It is especially acceptable, as it is a well-known fact that this branch of practice far excels in this country. The volume contains articles, by the editor, on "Diseases of the Vulva." These important subjects are well presented. This and the labor of editing, which is no small one, are performed in such a way as to reflect great credit on our fellow-townsman. The articles of Dr. Jenks, on the "Historical Sketch of American Gynecology;" "The Development of the Female Genitals," by Henry J. Garrigues;" "The Anatomy of the Female Pelvic Organs," by Henry C. Coe; "The Malformations of the Female Organs," by Henry J. Garrigues; "Gynecological Diagnosis," by Egbert H. Grandin; "General Consideration of Gynecological Surgery," by E. C. Dudley; "General Therapeutics," by Alexander J. C. Skene; "Electricity in Gynecology," by Alphonse D. Rockwell; "Menstruation and its Disorders," by W. Gill Wylie; "Sterility," by A. Reeves Jackson; "Diseases of the Vulva," by Mathew D. Mann;

"The Inflammatory Affections of the Uterus," by C. D. Palmer; "Subinvolution of the Vagina and Uterus," by Thaddeus A. Reamy; "Periuterine Inflammation," by Richard B. Maury; "Pelvic Hæmatocele and Hæmatomata," by Ely Van De Warker, are well and concisely written. By reading the titles of the articles, it will be seen that the first volume is of great value in this most important department of medicine. We congratulate the publishers on the most excellent production.

